# Feasibility of the “OA Coach” Mobile App to Support Individuals with Osteoarthritis: Development and Usability Testing

**DOI:** 10.2196/84857

**Published:** 2026-07-15

**Authors:** Vicky Duong, Naomi Bloul, Jocelyn Bowden, Karen Bracken, Kate Bryce, Leticia Deveza, Jillian Eyles, Abdolhay Farivar, Robin Huang, Sarah Kobayashi, Na Liu, Carin Pratt, Charlotte Strong, Venkatesha Venkatesha, Shirley Yu, David J Hunter

**Affiliations:** 1 Sydney Musculoskeletal Research Centre, The Kolling Institute, The University of Sydney, Level 10 Kolling Building, Reserve Road, St Leonards, New South Wales, 2065, Australia; 2 Department of Rheumatology, Royal North Shore Hospital, St Leonards, Australia; 3 Vulsen, Sydney, Australia; 4 Business Information Systems, The University of Sydney Business School, The University of Sydney, Sydney, Australia; 5 Department of Physiotherapy, Royal North Shore Hospital, St Leonards, Australia; 6 Research Directorate, Northern Sydney Local Health District, Royal North Shore Hospital, St Leonards, Australia

**Keywords:** mobile app, osteoarthritis, digital health, physical activity, self-management

## Abstract

**Background:**

The OA Coach mobile app was developed to support individuals with knee osteoarthritis in self-managing their condition. The app aims to fill a current gap in the osteoarthritis mobile app field by combining key features such as symptom tracking, objective activity tracking, educational modules, and encouragement notifications underpinned by behavior change theory.

**Objective:**

The aim of this study was to describe the development of the OA Coach mobile app and assess its usability in a 6-week feasibility study.

**Methods:**

The app was designed in consultation with consumers, rheumatologists, physiotherapists, and osteoarthritis researchers. The app prototype contained four screens: (1) a home screen to track goals and activities, (2) a progress page, (3) a learning page with self-directed modules, and (4) an inbox for communication with the study team. For the feasibility study, 30 participants were recruited between March and April 2024 from a database of osteoarthritis trial participants or through the Osteoarthritis Chronic Care Program at Royal North Shore Hospital, Sydney, Australia. Participants were eligible if they were aged 45 years or older, had knee pain ≥4 on an 11-point numerical pain rating scale and knee stiffness lasting <30 minutes in duration, or stiffness >30 minutes and diagnosed with knee osteoarthritis through a health care provider or radiographs. Participants were provided access to the app and asked to interact with it daily for 6 weeks. Outcomes were assessed through online questionnaires or through mobile app data. The primary outcome was usability, assessed using the mHealth App Usability Questionnaire (MAUQ). Secondary outcomes included computer self-efficacy and osteoarthritis knowledge. The quantitative data were summarized descriptively. Qualitative feedback was collected through open-ended survey responses and discussed within the research team to improve the app.

**Results:**

A total of 30 participants completed the study. There was a 1:1 ratio of male to female participants, with an average age of 66.9 (SD 9.1) years and a mean pain level of 6.0 (IQR 5.0-6.8) on an 11-point numerical pain rating scale. Twenty-nine responses from the MAUQ were available for analysis. Most statements scored >5 out of 7 (“somewhat agree”), indicating that the app was easy to use. The mean satisfaction score for the app on the MAUQ was 4.7 (SD 2.0) out of 7. Qualitative feedback from participants indicated the need for clear instructions on how to use and navigate the app, improved structure and integration of the exercise program, and improved tailoring of osteoarthritis education and support.

**Conclusions:**

Overall, the OA Coach app was well accepted by participants. Based on participant feedback, the app will be revised to improve aspects of clarity, ease of use, and personalization. The updated app will be tested against other methods of care delivery in a randomized controlled trial.

## Introduction

Osteoarthritis is the most common form of arthritis and a leading cause of disability worldwide. With the current population growth and aging, the number of Australians living with osteoarthritis is expected to increase, impacting over 3.11 million by the year 2040 [1]. There is no cure for osteoarthritis, and the current management strategies focus on alleviating symptoms. The core treatments for osteoarthritis include education for self-management, physical activity, and exercise [2,3]. These core treatments are appropriate for all individuals with osteoarthritis, regardless of the severity of their symptoms [2].

Many people with osteoarthritis do not receive appropriate care [4]. For example, surgical referral is often prioritized over nondrug treatments, despite conservative, nondrug care being strongly recommended in international guidelines for osteoarthritis management [4-6]. The use of digital health, combined with behavior change techniques (BCTs), may help to improve access to appropriate osteoarthritis care. BCTs (eg, feedback, self-monitoring, and reinforcement) are observable, replicable, and irreducible components of interventions designed to alter or redirect causal processes that regulate behavior [7]. Physical activity interventions that use BCTs are more effective in chronic disease populations [8], and physical activity interventions that include BCTs through digital health have been shown to increase physical activity in people with osteoarthritis in the short term [9]. Digital health is broadly defined as the use of technologies such as wearables, mobile phones or apps, and websites for the treatment, prevention, and maintenance of health [10]. The World Health Organization has identified digital health as a global priority. Greater adoption of digital health initiatives will support accessible, equitable, and universal access to quality health services, as well as strengthen and scale up health promotion, disease prevention, management, and rehabilitation [11]. The COVID-19 pandemic has further accelerated the use of these technologies, and digital health is being increasingly used for the management of knee osteoarthritis [12-16]. Compared to traditional care, the use of digital health care has provided similar improvements in pain and function in osteoarthritis and back pain populations (systematic review of 23 studies, n=4994 participants) [17]. Our research group also found similar results in individuals following knee replacement (systematic review of 17 studies, n=2188 participants) and in a recent randomized controlled trial (n=102 participants) where participants received a combination of digital interventions (exercise mobile app, activity tracker, and online health coaching) [15,18]. In Australia, the majority of households have access to the internet (88%), even in remote and very remote settings (77%). Of those with internet access, 91% have access to a laptop, desktop, or smartphone [19], highlighting the potential to scale up effective digital interventions for osteoarthritis management. Access to evidence-based osteoarthritis care through a digital mobile health app could be a potential solution for low-cost and accessible care.

Mobile apps designed to support the management of osteoarthritis exist; however, no osteoarthritis-specific app has measured or monitored physical activity objectively [20]. As increasing and maintaining physical activity levels is a core aspect of osteoarthritis management, it is important that reliable measures of physical activity are incorporated into these apps. Previous studies have suggested that participants tend to inaccurately report their adherence to exercise and physical activity during home-based programs [21,22]. A recent systematic review also found that existing mobile apps for osteoarthritis management lack quality information, structured exercise programs, and scientific testing [20].

We developed a mobile app called OA Coach to support individuals with osteoarthritis in self-managing their condition. OA Coach aims to fill a current gap in the osteoarthritis mobile health field by combining key features such as symptom tracking, objective activity tracking (step count and sleep), tailored educational modules, and encouragement notifications, all underpinned by behavior change theory and developed with experts in osteoarthritis. The primary aim of this paper was to outline the development of the OA Coach mobile app and examine the usability of the app in participants with knee osteoarthritis in a 6-week feasibility study. Secondary outcomes include changes in knee symptoms, physical activity, sleep, feelings of depression, anxiety, stress, and osteoarthritis knowledge after app use.

## Methods

### Mobile App Prototype Development

The OA Coach app was co-designed with a multidisciplinary team including osteoarthritis researchers, rheumatologists, physiotherapists, and consumers living with osteoarthritis. The app prototype was developed by Vulsen, a developer with expertise in developing mobile apps for chronic disease management [23,24]. The study team (VD, NB, JE, SK, DJH) tested the mobile app prototype for approximately 2 weeks. A 1-hour co-design workshop was also held with 6 consumers with lived experience of osteoarthritis to gather initial feedback regarding the features and visual aspects of the app prototype. Co-design participants were given access to the mobile app to download onto their personal devices 1 week prior to the workshop. In the workshop, a brief overview of the app was presented, followed by the participants mapping likes or dislikes and potential areas for improvement for the app and a group discussion. Following alpha-testing, issues with the functionality or content of the app were relayed to the developer for resolution prior to the 6-week feasibility study.

### Mobile App Features and Functionality

**Figure 1.**
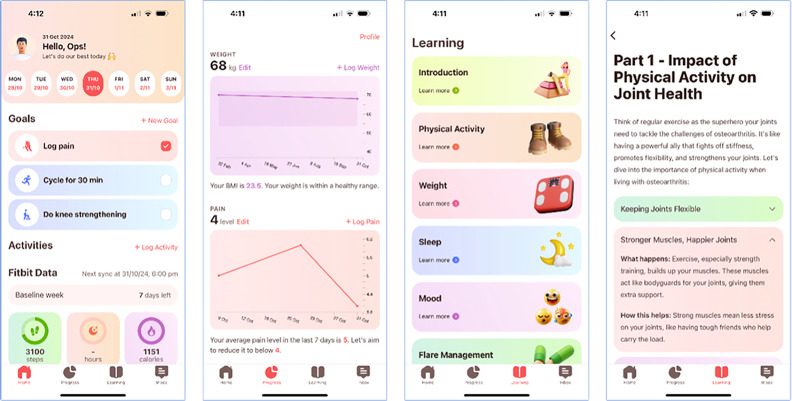
Overview of the OA Coach mobile app’s Home, Progress, and Learning pages.

Details on the main features of the app are summarized in Textbox 1. An overview of the Home, Progress, and Learning pages is displayed in Figure 1. The app’s log-in page required participants to enter their date of birth and answer questions about their knee pain (pain severity, stiffness duration). This information was used to make a clinical diagnosis based on the National Institute for Health and Care Excellence guidelines [25], which then determined access to the app. Following this, participants were prompted to complete questions about their weight, height, sleep, mood, and current activity levels in order to trigger corresponding educational modules targeted to the users’ needs. For example, if a participant’s BMI was above a healthy range or the questionnaire responses indicated poor sleep, participants would be assigned the “Weight Management” or “Sleep” modules, respectively.

Textbox 1. **Functionalities associated with each feature of the OA Coach app prototype.**

**Home screen**

- Participants prospectively set personal goals (eg, related to physical activity, exercise, meditation) during the registration process of the app, which are displayed as a checklist on the home screen under Goals.
- Questionnaires for mood and sleep, and logging of pain scores, will also appear under Goals.
- Fitbit data, including the number of steps, hours slept, and number of calories burned, are displayed.

**Progress**

- Weight is tracked in kilograms. BMI is calculated with the user’s height inputted at registration and displayed at the bottom of the graph. If the user’s BMI is between 18.5 and 24.9 kg/m^2^, it will display “Your weight is within a healthy range.” Otherwise, the graph will say “Let’s aim to reach a healthy weight range between X kg and X kg.”
- Knee pain over the past 24 hours is tracked on a 0‐10 numerical rating scale, where 0 represents no pain and 10 represents extreme pain. The average pain over the past 7 days is displayed at the bottom of the graph.
- Sleep and step count data are automatically populated with Fitbit data.

**Learning**

- Seven learning modules related to the core aspects of osteoarthritis management are displayed (introduction to osteoarthritis, physical activity, weight, sleep, mood, osteoarthritis flare management, and exercise). Each module, apart from physical activity, has 3 units: “Educational resources: Learn the basics, Self-guided help: Digging deeper, and Expert help: Getting more help.”
- If participants experience an episode of a pain flare, they will be prompted by a notification to complete the Flare Management module. Similarly, if they have an episode of low mood or sleep, they will be prompted to complete the Mood or Sleep modules, respectively. These modules will appear initially as a pop-up notification and then on participants’ Home page in the Goals section.

**Messaging**

- Initially set up for study staff to send one-way messages to participants related to their progress. The messages are sent from a custom dashboard and appear for participants in the messaging tab, which displays messages in a format similar to text messages on a mobile phone. Participants will also receive a push notification on their phone home screen alerting them to the new message.

Exercise plans for the app were displayed under the “Learning” page. The page included a brief introduction to knee-strengthening exercises, including the benefits of exercise, how to complete the exercise programs (warm up, joint specific exercises, cardiovascular endurance exercises [if possible], warm down), how to assess difficulty and progression, and frequency of program. Separate plans for individuals with good mobility, moderate mobility, and limited mobility were presented, with 3 progressions within each plan. Each exercise plan included instructional photos and descriptions as well as suggested dosage. Participants were free to choose a plan which best aligned with their current mobility status.

Participants were also provided with an activity tracker to wear for the duration of the study. The app requires that participants wear the Fitbit tracker to collect baseline data on sleep and physical activity levels in order to generate appropriate goals. The step and sleep count data are displayed on the “Home” and “Progress” pages of the app. The generated goals are displayed under the “Goals” heading on the “Home” page.

### Behavior Change Theory

Behavior change theory was used to help motivate users to increase their physical activity and sleep levels, as well as complete tasks such as logging pain scores or completing surveys. BCTs, such as goal setting, feedback on behaviors, and graded tasks, were integrated into the setup process and push notifications. Participants were asked to set goals during the initial setup process of the app, where they were asked about their current exercise and physical activity levels in a questionnaire. Their responses were used to create weekly goals. For example, if a participant enjoyed playing a sport once a week, a goal would automatically be created to increase the frequency of the sport to align with the World Health Organization physical activity guidelines for older adults [26]. Feedback on behaviors, as well as graded tasks, was provided to participants as push notifications for meeting their step goal at the end of the day. Self-monitoring of pain levels and body weight was also encouraged through notifications and logged in the “Progress” page of the app, along with sleep and step count data. Further details of various OA Coach features mapped to the Behavior Change Taxonomy are outlined in Multimedia Appendix 1.

### Feasibility Study Design

The feasibility study involved providing the OA Coach mobile app to study participants for 6 weeks to assess the usability of the developed app. The protocol for the feasibility study was registered on the Australian and New Zealand Clinical Trials Registry before commencement (registry number: 12623001223628). The results of this study are reported in accordance with the CONSORT (Consolidated Standards of Reporting Trials) checklist (Checklist 1).

### Ethical Considerations

Ethical approval was granted by the Northern Sydney Local Health District Human Research Ethics Committee (2023/ETH02288). Participant eligibility was assessed using an online survey, and participants who passed the screening survey provided electronic consent through a Research Electronic Data Capture e-consent form.

### Participants

Potentially suitable participants were identified by rheumatologists and physiotherapists within the Osteoarthritis Chronic Care Program at Royal North Shore Hospital, Sydney, Australia, and were provided with a link to the eligibility screening survey. Participants from past clinical trials who had consented to be contacted about future research studies were also emailed and invited to participate in the study.

Individuals were eligible for the study if they were aged 45 years or older, had knee pain ≥4 out of 10 on an 11-point Numerical Pain Rating Scale (NPRS; where 0 represents “no pain” and 10 represents “worst imaginable pain”), and experienced knee stiffness lasting no longer than 30 minutes after rising from bed in the morning. Participants were also eligible to participate in the study if they had knee stiffness >30 minutes but had been diagnosed with knee osteoarthritis through radiographs or a health care practitioner. This inclusion criterion was added as an amendment to the study, as a number of participants with a confirmed diagnosis of osteoarthritis were being excluded due to stiffness slightly exceeding 30 minutes. These participants were contacted either from either our database of previous participants or were recruited from the Osteoarthritis Chronic Care Program at Royal North Shore Hospital thus already having a confirmed diagnosis of osteoarthritis. Participants also required ownership of a smartphone with internet access in order to download the OA Coach app. The exclusion criteria for the study were as follows: a diagnosis of inflammatory arthritis, such as rheumatoid arthritis or gout, and any comorbidities or medical conditions that would prevent participation in the physical activity component of the app.

### Intervention

All participants received a Fitbit tracker (Fitbit Inspire 2) in the post and instructions on how to download the OA Coach app on their personal smartphones and link the Fitbit tracker. The OA Coach app is freely available on app stores; however, its access remained password-protected during the evaluation period. Participants were instructed to wear the Fitbit tracker for a baseline period of 1 week and were then asked to interact with the app daily for 6 weeks. Participants were instructed to contact the study coordinator through email if they experienced any technical issues in setting up the OA Coach app or Fitbit tracker. The study coordinator aimed to resolve any technical issues through email, prior to contacting participants by phone.

### Outcomes

Three sets of data were obtained from the study including baseline and 6-week online questionnaires, data from the mobile app (eg, step count, hours slept, the 21-item Depression, Anxiety and Stress Scale [DASS-21] and Pittsburgh Sleep Quality Index [PSQI] questionnaires), and qualitative feedback from surveys at 6 weeks.

### Primary Outcome

The mHealth App Usability Questionnaire (MAUQ) for Standalone mHealth Apps Used by Patients was used to assess the usability of the OA Coach app for the primary outcome [27]. The questionnaire consists of 18 statements, and participants were asked to rate their agreement with each statement on a 7-point Likert scale, ranging from strongly disagree to strongly agree [27]. To determine the usability of the app, the total score was calculated and averaged across responses, with higher scores representing higher usability.

### Secondary Outcomes

The Modified Computer Self-Efficacy Scale (mCSES) and the Osteoarthritis Knowledge Scale (OAKS) were used to assess technology self-efficacy and osteoarthritis knowledge, respectively, at baseline and 6 weeks through surveys. The mCSES asks respondents to rate their confidence using a new technology on a scale from 1 to 10, where 1 represents “not confident at all” and 10 “totally confident” [28]. The OAKS is an 11-item scale assessing important knee osteoarthritis knowledge, with scores ranging from 11 to 55, with higher scores indicating greater knowledge about osteoarthritis [29].

Secondary outcomes collected through the app included changes in knee pain (NRS), BMI, mood, sleep quality and disturbances, physical activity (average step count over the last 7 days), and time asleep (average sleep duration over the last 7 days). The DASS-21 was used to measure the emotional states of depression, anxiety, and stress. The questionnaire consists of 21 statements, with 7 statements pertaining to each subscale. Participants were asked to rate their agreement with each statement from 0 to 3 (where 0 indicates “Never,” and 3 indicates “Almost always”) [30]. Scores for each subscale were summed and multiplied by 2 for interpretation. The PSQI is a 19-item self-reported questionnaire assessing various aspects of sleep quality (eg, duration, latency, disturbances) over the past month. Scores for each question range from 0 to 3, with higher scores indicating more acute sleep disturbances [31].

Knee pain (NRS) and body weight (kg) were self-reported by participants. Physical activity and time asleep were automatically collected from participants’ Fitbit devices, which synced to the mobile app.

### Qualitative Feedback

Participants were also asked to provide any feedback regarding the app through open text fields with the option of including their contact details should they wish to discuss the feedback in person with a study team member.

The outcome measures collected in this study were exploratory in nature and were not used for progression, modification, or stopping decisions within the study protocol.

### Sample Size

As this study was a feasibility study, no formal sample size calculations were conducted. We aimed to recruit a total of 30 participants for this study, which is an acceptable size for a feasibility study [32].

### Statistical Analysis

Participant baseline demographics and clinical characteristics were expressed using means and SDs or medians and IQRs if not normally distributed. The primary outcome (MAUQ) was summarized descriptively. The secondary outcome measures at baseline and 6 weeks were summarized using median and IQR. The change scores (baseline minus 6 weeks) were summarized using median and IQR. Analyses were conducted using STATA (version 18.0). Qualitative data from participant feedback were collated into an Excel spreadsheet, and each comment was mapped to a general category (eg, App download, Fitbit integration, goals, instructions, learning materials, notifications, design, or other) by 1 researcher (NB). These were reviewed by a second researcher (VD) and suggestions on how to improve the app were discussed and documented. The suggestions were then reviewed within the wider study team, including the app developer. Changes that the researchers or developer deemed reasonable and appropriate informed the next iteration of the OA Coach mobile app. Changes that required additional resources and funding were temporarily put on hold.

## Results

### Participant Demographics

A total of 30 participants were recruited between March 2023 and April 2023. There was an even split of female and male participants (n=15 per sex), and participants had an average age of 66.9 (SD 9.1) years and a median pain level of 6.0 (IQR 5.0-6.8) on an 11-point NRS. The participant baseline demographics are summarized in Table 1. Due to technical issues, there were some participants who did not have complete step, and sleep data at baseline and demographics in Table 1 are based on a complete case analysis.

**Table 1.** Participant baseline demographics.

Characteristics	Values (n=30)
Age (y), mean (SD)	66.9 (9.1)
Female, n (%)	15 (50)
NRS^a^ pain, median (IQR)	6.0 (5.0-6.8)
Technology self-efficacy (mCSES)^b^, median (IQR)	89.0 (74.0-99.0)
Osteoarthritis knowledge (OAKS)^c^, median (IQR)	41.0 (38.0-49.0)
BMI (kg/m^2^), median (IQR)	26.7(24.4-31.2)
Depression (DASS-21)^d^, median (IQR)	6.0^e^ (2.0-8.0)
Anxiety (DASS-21), median (IQR)	4.0^e^ (0.5-7.0)
Stress (DASS-21), median (IQR)	10.0^e^ (4.0-14.0)
Sleep quality and disturbances (PSQI)^f^, median (IQR)	6.5 (5.0-10.3)
Physical activity^g^, median (IQR)	8073^h^ (5636-10642)
Time asleep^i^, median (IQR)	7.0^j^ (5.6-7.7)

a NRS: Numerical Rating Scale.

b mCSES: Modified Computer Self-Efficacy Scale.

c OAKS: Osteoarthritis Knowledge Scale.

d DASS-21: 21-item Depression, Anxiety, and Stress Scale.

e Twenty-seven participants.

f PSQI: Pittsburgh Sleep Quality Index.

g Average step count over the last 7 days.

h Twenty-nine participants.

i Average sleep duration in hours over the last 7 days.

j Twenty-four participants.

### Primary Outcome

Overall, participants found the app usable, with the majority of scores greater than 5 out of 7 on the MAUQ. Most participants found the app easy to use (mean 5.8, SD 1.5), easy to learn how to use (mean 6.1, SD 1.2), and were satisfied with the amount of time involved in using the app (mean 6.0, SD 0.9). The lowest scoring statements were regarding improving access to health care services (mean 3.6, SD 1.8) and the app functionalities and capabilities (mean 4.1, SD 1.9), which are in alignment with the qualitative data collected. The results of the MAUQ statements are summarized in Table 2.

**Table 2.** Results of the mHealth App Usability Questionnaire (MAUQ).

Statement^a^	Mean (SD)^b^	Min-max
The app was easy to use.	5.8 (1.5)	2-7
It was easy for me to learn to use the app.	6.1 (1.2)	2-7
The navigation was consistent when moving between screens.	5.5 (1.5)	1-7
The interface of the app allowed me to use all the functions (such as entering information, responding to reminders, viewing information) offered by the app.	5.1 (1.9)	2-7
Whenever I made a mistake using the app, I could recover easily and quickly.	5.2 (1.7)	0-7
I like the interface of the app.	5.1 (1.7)	2-7
The information in the app was well organized, so I could easily find the information I needed.	5.2 (1.7)	2-7
The app adequately acknowledged and provided information to let me know the progress of my action.	5.3 (1.6)	2-7
I feel comfortable using this app in social settings.	5.8 (1.6)	1-7
The amount of time involved in using this app has been fitting for me.	6.0 (0.9)	4-7
I would use this app again.	5.2 (1.7)	2-7
Overall, I am satisfied with this app.	4.7 (2.0)	0-7
The app would be useful for my health and well-being.	5.4 (1.8)	0-7
The app improved my access to health care services.	3.6 (1.8)	0-7
The app helped me manage my health effectively.	5.1 (1.2)	2-7
This app has all the functions and capabilities I expected it to have.	4.1 (1.9)	1-7
I could use the app even when the internet connection was poor or not available.	4.3 (1.9)	0-7
This mHealth^c^ app provides an acceptable way to receive health care services, such as accessing educational materials, tracking my own activities, and performing self-assessment.	5.3 (1.2)	3-7

a n=29; 1 participant had missing data.

b Scores range from 1 to 7, where 1 indicates strongly disagree, 2 indicates disagree, 3 indicates somewhat disagree, 4 indicates neither agree nor disagree, 5 indicates somewhat agree, 6 indicates agree, and 7 indicates strongly agree.

c mHealth: mobile health.

### Secondary Outcomes

The NRS median pain reduced from a median of 5.0 (IQR 4.0‐6.0) at baseline to 4.0 (IQR 2.0‐5.0) at 6 weeks, giving a within-patient median reduction of 1 point (IQR 0.0‐3.0). The modified Computer Self-Efficacy Scale (mCSES) moved in the opposite direction: the median score declined from 89 (IQR 74‐100) to 81 (IQR 67‐93), reflecting a median change (reduction) score of 2.5 points (IQR –1.0 to  7.0). The OAKS score did not indicate a substantial change in knowledge (median change of 0.0 points, IQR –2.0  to  3.0).

The magnitude of change in BMI was very small—change from a median 26.7 (IQR 24.4‐31.2) to 26.6  (IQR 24.5‐29.1)—corresponding to a median within-patient change of 0.02 (IQR –0.15  to  0.05). There was a small improvement in the psychological distress measure. The median depression scores falling from 6.0 (IQR 2.0‐8.0) to 4.0 (IQR 2.0‐8.0), anxiety from 4.0 (IQR 0.0‐6.0) to 2.0 (IQR 2.0‐6.0), and stress from 10.0 (IQR 4.0‐16.0) to 6.0 (IQR 2.0‐12.0). Sleep quality improved slightly, with the median PSQI score decreasing from 6.5 (IQR 5.0‐10.5) to 6.0 (IQR 4.0‐9.0).

The median 7-day step count increased from 8073 (IQR 5392‐10,726) to 8616 (IQR 6028‐10,057), a median gain of 446 steps (IQR –1433  to  2,322), while the average nightly sleep duration remained almost equal at median 7.02 (IQR 5.56‐7.68) hours and 6.90 (IQR 5.40‐7.65) hours. These changes are minimal and may be partially explained by the cohort that was recruited. Most participants were either previous participants of our clinical trials or had been receiving care from an osteoarthritis management team and therefore were more likely to be knowledgeable about their condition, already be engaged in some form of physical activity and have sufficient sleep hygiene. Secondary outcomes are summarized in Table 3.

**Table 3.** Secondary outcomes.

Measure	Baseline, median (IQR)	Six weeks, median (IQR)	Change (baseline to 6 weeks), median (IQR)
NRS^a^ pain	5.0 (4.0 to 6.0)	4.0 (2.0 to 5.0)	1 (0.0 to 3.0)
Technology self-efficacy (mCSES)^b^	89.0 (74.0 to 100.0)	81.0 (67.0 to 93.0)	2.5 (–1.0 to 7.0)
Osteoarthritis knowledge (OAKS)^c^	41.0 (38.0 to 49.0)	42 (35 to 48.0)	0.0 (–2.0 to 3.0)
BMI (kg/m^2^)	26.7 (24.4 to 31.2)	26.6 (24.5 to 29.1)	–0.02 (–0.15 to 0.05)
Depression (DASS-21)^d^	6.0 (2.0 to 8.0)	4.0 (2.0 to 8.0)	0.0 (0.0 to 6.0)
Anxiety (DASS-21)	4.0 (0.0 to 6.0)	2.0 (2.0 to 6.0)	0.0 (–2.0 to 2.0)
Stress (DASS-21)	10.0 (4.0 to 14.0)	6.0 (2.0 to 12.0)	2.0 (–6.0 to 6.0)
Sleep quality and disturbances (PSQI)^e^	6.5 (5.0 to 10.5)	6.0 (4.0 to 9.0)	1.0 (0.0 to 2.0)
Physical activity^f^	8073 (5392 to 10726)	8616 (6028 to 10057)	446 (–1433 to 2322)
Time asleep^g^	7.02 (5.56 to 7.68)	6.9 (5.4 to 7.65)	0.15 (–0.58 to 0.57)

a NRS: Numerical Rating Scale.

b mCSES: Modified Computer Self-Efficacy Scale.

c OAKS: Osteoarthritis Knowledge Scale.

d DASS-21: 21-item Depression, Anxiety, and Stress Scale.

e PSQI: Pittsburgh Sleep Quality Index.

f Average step count over the last 7 days

g Average sleep duration in hours over the last 7 days

Overall, when comparing outcomes at 6 weeks to baseline, there was a small reduction in pain and psychological distress; slight improvements in sleep quality and physical activity; and stability in knowledge, BMI, and sleep duration. These outcomes were expected in a small, uncontrolled feasibility study that was not designed to detect statistically significant changes within the group.

### Qualitative Feedback

The qualitative feedback largely focused on usability and suggestions for improving the app, such as better integration of the exercise programs and improved personalization of goals and activity tracking. Additionally, participants highlighted the need for improved onboarding support. Participants also noted confusion over the labeling of certain features. The main areas for improvement and updates made to the next iteration of the OA Coach app are outlined in Table 4.

**Table 4.** Summary of qualitative feedback from participants at 6 weeks.

Page	Areas lacking and suggestions for improvement	Updates made to OA Coach app
Goals	Not possible to set a one-off goal in the app, only recurring goals Tying goals to specific time was not appropriate Unable to find DASS-21^a^ and PSQI^b^ questionnaires	A new “Activities” section was created to log one-off activities. The goals section will remain for recurring goals. Clarified that the time next to the goal was for the time for the reminder to be sent. The time has been removed from under the goal so as not to cause confusion but can be edited by selecting the icon next to the goal. DASS-21 and PSQI questionnaires were renamed to “Sleep” and “Mood” questionnaires to avoid confusion. Clarified instructions on how to complete the questionnaires and goals to fill questionnaires will repeat daily until they have been completed.
Instructions	Setting up Fitbit goals and duration of exercise activities were confusing	Clearer instructions and explanatory text were provided. An introductory video was created to explain all features of the app.
Learning	Some links in the article did not work Could not find the knee strengthening exercises	Links in articles were checked and updated. An introductory video was created to explain how to use the app and features.
Messages	Feedback notifications were too generic	Notifications were modified, and changes were made underpinned by behavior change theory. New notifications and messages underwent comprehensive co-design and survey study.

a DASS-21: 21-item Depression, Anxiety, and Stress Scale.

b PSQI: Pittsburgh Sleep Quality Index.

## Discussion

The OA Coach mobile app is designed to provide education to people with osteoarthritis to help them self-manage their condition. The app aims to address the current gaps in mobile apps for the management of osteoarthritis, focuses on all aspects of osteoarthritis management (eg, pain flares, weight management, mood, sleep, physical activity, and exercise), and has been co-designed with end user input. Overall, the usability of the OA Coach mobile app prototype was deemed acceptable by participants when assessed using the MAUQ. The lowest scoring item on the MAUQ was access to health care services, and although the app provides recommendations for how to access services in the learning modules, including the types of support available with relevant links, improvements to the app could be made to make this more explicit, for example, including a directory of health care professionals or service providers who manage different aspects of osteoarthritis and their contact details. The secondary outcomes demonstrated small reductions in pain and psychological distress and slight improvements in sleep quality and physical activity (step count). Interestingly, technology self-efficacy worsened. However, as this was a feasibility study only, the sample size was not powered to reliably detect a true effect. The 6-week duration of the study was also likely too short to detect a change in outcomes.

From the qualitative feedback, study participants recommended improving the clarity and personalization aspects of the OA Coach mobile app. As a result, the app was updated to include more instructions and clarification text within different features of the app. Updates to the app were made, including the addition of an introductory instructional video and an improved interface for the exercise programs and “Inbox.” A total of 80 notifications sent through the app also underwent an extensive co-design process and were refined. An additional 12 educational messages were also co-designed for the new “Inbox” feature of the app [33].

A major strength of the study is that the OA Coach mobile app was co-designed and tested with people with lived experience of osteoarthritis. There was also input from researchers and health professionals with various backgrounds and expertise. The developer, Vulsen, is familiar with the requirements for data privacy in Australia, and their mobile apps have been approved for use in collecting patient data within local health districts within Sydney, Australia. Another major strength of the study is that we used objectively measured physical activity and sleep to provide tailored recommendations in the app. We also used validated questionnaires for mood and sleep, allowing the app to tailor learning modules on these topics to participants. The balanced distribution of female to male participants was also a strength of our study, as there is usually a greater proportion of female participants in osteoarthritis clinical trials [34].

Limitations of our study include selection bias—we were only able to include participants in the study who owned a smartphone and therefore may be more familiar with the use of mobile apps. The usability scores of the app may be biased upward due to the self-selection of participants who were more technology-literate. The participants in our trial were generally older as well and living in metropolitan Sydney, which limits the generalizability of the results. Participants were also recruited from our database of previous clinical trial participants or from the Osteoarthritis Chronic Care Program. As a result, they were more likely to have prior knowledge about their condition and to already be engaging in some form of physical activity or exercise. At baseline, participants were already achieving an adequate level of physical activity as measured by step count and were sleeping the recommended number of hours. This high starting point limits the potential for further improvement, which may partially explain why changes in secondary outcomes were minimal. The study had a short follow-up duration of only 6 weeks, and a longer period of follow-up (eg, 12 wk) would have been ideal to evaluate long-term outcomes and behavior change. For the purposes of this study, we did not analyze the app meta-data, such as time spent interacting with the app and completing learning modules; however, these data may be used to refine future versions of the app. Finally, the educational content featured in our app is mainly limited to knee osteoarthritis; future versions of the app may also target other commonly affected joints, such as the base of the thumb or the hip joint.

The OA Coach mobile app was theory-driven and informed by behavior change theory and best-evidenced osteoarthritis care. The components of the app extend beyond aggregating features and are intended to enhance users’ capability, motivation, and self-efficacy. The OA Coach app combines the core aspects of osteoarthritis management, alongside objective monitoring of activity, symptoms, and behaviors. To our knowledge, this is the first mobile app developed for knee osteoarthritis management in the Australian market, which has the ability to measure physical activity objectively using a fitness tracker. Our methodology was consistent with a systematic review published in 2019 by Choi et al [35] that provided a framework for developing mobile apps for osteoarthritis management, particularly apps that have the ability to track pain and symptoms, provide instructional audio/video of exercise regimes, and set goals to maximize adherence. A more recent review by Hensley et al [20] in 2023 also found a lack of quality information, structured exercise programs, and health information protection in the mobile apps evaluated (n=14). OA Coach addresses many of the features missing in previous mobile apps developed for osteoarthritis management. The app will also undergo effectiveness testing in a 3-armed randomized controlled trial before being launched in public app stores [36]. This will also include in-depth interviews with participants to discuss barriers and facilitators to using the app. This will ensure that the final version of the app is effective at managing osteoarthritis-related symptoms. Future updates to the OA Coach mobile app functionality will be informed by consumer feedback as well as data analytics to ensure that the app continues to meet the evolving needs, preferences, and expectations of people living with osteoarthritis.

The results from this feasibility study provide evidence to support the use of the OA Coach mobile app by encouraging patients to self-manage their condition by providing educational resources; addressing comorbidities, such as sleep and mood changes that commonly coexist with osteoarthritis; and encouraging them to be more physically active. The feedback from the feasibility study has been used to refine the current version of the OA Coach app, and it is currently being tested in a randomized controlled trial assessing 3 different models of care. Further development of the app may involve the use of generative artificial intelligence to further tailor notifications to provide personalized recommendations, and the ability to sync with Apple Health or Google Health to track activity levels without the use of an activity tracker.

## Supplementary material

10.2196/84857 Multimedia Appendix 1 Mapping of OA Coach features to Behavior Change Taxonomy.

10.2196/84857 Checklist 1 CONSORT Checklist

## Abbreviations

BCT: behavior change technique
CONSORT: Consolidated Standards of Reporting Trials
DASS-21: 21-item Depression, Anxiety, and Stress Scale
MAUQ: mHealth App Usability Questionnaire
mCSES: Modified Computer Self-Efficacy Scale
NRS: Numerical Rating Scale
OAKS: Osteoarthritis Knowledge Scale
PSQI: Pittsburgh Sleep Quality Index
