# Supplementary material for: Feasibility of the “OA Coach” Mobile App to Support Individuals with Osteoarthritis: Development and Usability Testing
Source: JMIR Hum Factors. 2026 Jul 15;13:e84857. doi: 10.2196/84857 (PMC13372266; doi:10.2196/84857)
Supplement: Multimedia Appendix 1 [file humanfactors-v13-e84857-s001.docx]

Multimedia Appendix 1: Mapping of OA Coach features to Behaviour Change Taxonomy

| No. | Label | Example of OA Coach feature |
| --- | --- | --- |
| 1.1 | Goal setting (behaviour) | The app has the ability to set Meditation, Exercise or Custom goals within the app including frequency, days to display goal and notification time |
| 1.4 | Action planning | The app has the ability to change notification times of goals to prompt planning of certain activities e.g. can change timing of Walking notification to lunch time, where user is more likely to have time to walk |
| 2.2 | Feedback on behaviour | The OA Coach app syncs with a Fitbit device and  Data is displayed under “Activities” e.g. number of steps walked each day is displayed and participants will receive a notification if they reach their step goal |
| 2.3 | Self-monitoring of behaviour | Methods have been established to allow participants to record/track their behaviour  e.g. knee pain and weight, as well as steps and sleep |
| 2.4 | Self-monitoring outcomes of behaviour | Knee pain and body weight (as well as body mass index) are displayed on the Progress page. Healthy body mass index ranges based on participant height is displayed on the graph.  Sleep and steps are also displayed on the Progress page. |
| 4.1/6.1 | Instruction on how to perform a behaviour/Demonstration of the behaviour | Detailed instructions on how to perform knee specific exercises as well as instructional photos are provided in the Learning page under Exercise plans. |
| 5.1 | Information about health consequences | Learning modules provide written information about health consequences on performing the behaviour e.g. In the sleep module participants can read about the many benefits of getting good quality sleep |
| 7.1 | Prompts/cues | Motivational notifications have been designed to prompt certain behaviours e.g. Notifications are sent to participants to log their knee pain |
| 9.1 | Credible source | Verbal communication regarding different behaviours through the form of podcasts are linked in various Learning Modules. |
| 10.4 | Social reward | Participants are sent congratulatory notifications for completing their goals |
